# Retraction: Five active components compatibility of astragali radix and angelicae sinensis radix protect hematopoietic function against cyclophosphamide-induced injury in mice and t-BHP–induced injury in HSCs

**DOI:** 10.3389/fphar.2022.1051973

**Published:** 2022-09-28

**Authors:** Frontiers Editorial Office

The journal retracts the 22 August 2019, article cited above.

Following publication, concerns were raised regarding the substantial similarity of content with two of the authors’ previous publications. An investigation was conducted in accordance with Frontiers’ policies, whereby it was confirmed that the degree of reuse of the previously published content is inconsistent with our editorial requirements for originality. Following the Committee on Publication Ethics guidelines, this article, published most recently, is retracted. This retraction was approved by the Chief Editors of Frontiers in Pharmacology, and the Chief Executive Editor of Frontiers. The authors agree to this retraction.

The authors concur with the retraction and sincerely regret any inconvenience this may have caused to the reviewers, editors, and readers of Frontiers in Pharmacology.

